# Supplementary material for: Frameworks for estimating causal effects in observational settings: comparing confounder adjustment and instrumental variables
Source: BMC Med Res Methodol. 2023 May 22;23:122. doi: 10.1186/s12874-023-01936-2 (PMC10201752; doi:10.1186/s12874-023-01936-2)
Supplement: Supplementary file 1 — Additional file 1. More information on how the IVs were created as well as checking the relevance and sensitivity to unobserved confounders of these IVs. Additionally, we investigate diagnostics related to IPTW: pre- and post-matching balance as well as the positivity assumption. [file 12874_2023_1936_MOESM1_ESM.docx]

Supplementary Appendix

Tutorial in Biostatistics: Principles of Estimating Causal Effects in Observational Settings

Roy S. Zawadzki, Joshua D. Grill, Daniel L. Gillen, ADNI Investigators

Section 1: Selection and investigation of IVs

The four IVs were computed using the data. First, number of years between the FDA approval of donepezil on November 11^th^ 1996 and the subject’s study start date. Second, cardiovascular disorders that were specifically bradycardia, syncopal episodes, heart block and arrhythmia were found via keyword search on subject-reported conditions. Third, asthma, which was matched via keyword search. Fourth, GI disorders that were generally reported by a subject or specific conditions of GERD, diverticulitis, nausea, and irritable bowel syndrome.

Distributions for each treatment group for time since approval is visualized in Figure A1 and Table A1 shows the difference in proportions the binary instruments. For each binary IV, the proportion of compliers (i.e. difference of proportions) is small. The trimodality in Figure A1 is due to there being distinct waves of ADNI and, overall, the treatment groups are relatively indiscernible with respect to the shape of their distributions except for the region beyond 20 years since approval. Hence, we can reasonably conclude that our IVs are weak.

Table A1: Proportion of donepezil assignment by instrument level

|  | Donepezil (n = 134) | Control (n = 483) | Difference in proportions |
| --- | --- | --- | --- |
| Heart Disorder (n (%)) | 5 (3.7) | 11 (2.3) | 0.014 |
| Asthma | 5 (3.7) | 39 (8.1) | -0.044 |
| GI Disorder | 32 (23.9) | 136 (28.2) | 0.043 |

Figure A1: Histogram of relative frequency versus time since approval by treatment group


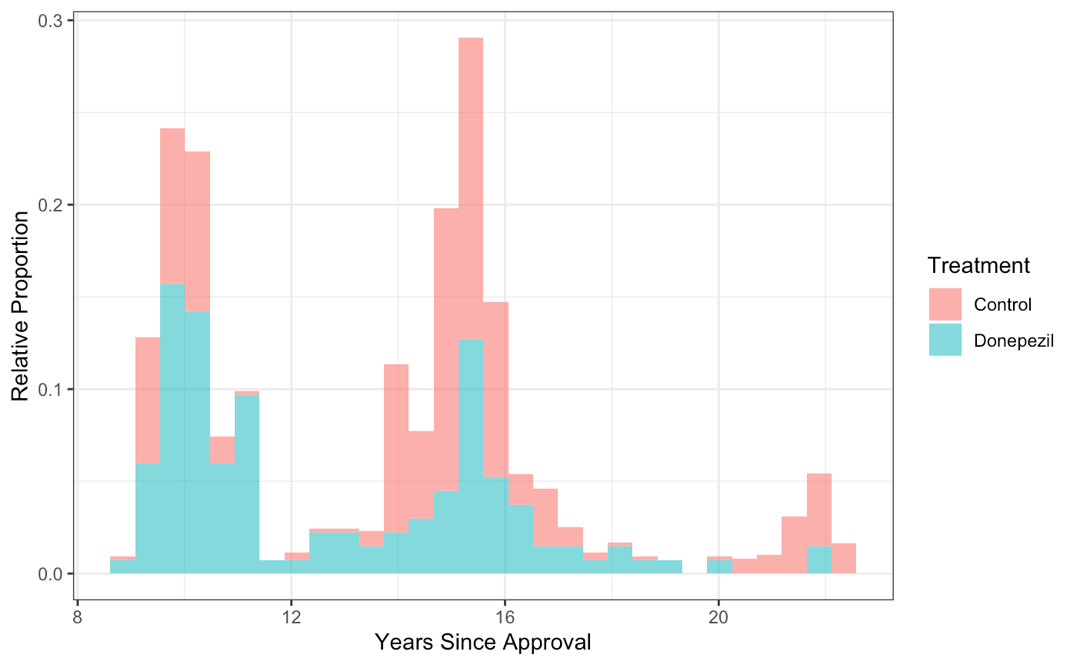


Table A2-4 check for any imbalance of confounders across levels of the binary IVs with the notion being that imbalance in an observed confounder suggests that there is an association between the IV and an unobserved confounder correlated with this observed confounder.^1^ We can see that there are a few variables that have high SMD such as APOE4 count for heart disorder and age for asthma. The imbalance of the non-cardiovascular comorbidities for asthma is also of concern. GI disorder does not appear to have any variables with large SMD but P-Tau is still sizable. We have evidence to conclude that the independence of these IVs may be violated but we will assume that the present confounders block many of the violating pathways that lead to the outcome (e.g. cardiovascular issues for heart disorder).

Table A2: Confounders stratified by presence of heart disorder IV

|  | Heart Disorder (n =16) | No Heart Disorder  (n = 533) | SMD^a^ |
| --- | --- | --- | --- |
| Cardiovascular Issues – Yes (n (%) | 16 (100.0) | 422 (70.2) | 0.921 |
| Neurological Issues – Yes (n (%)) | 10 (62.5) | 204 (33.9) | 0.596 |
| Renal Issues - Yes (n (%)) | 11 (68.8) | 266 (44.3) | 0.51 |
| Age (mean (SD) | 75.51 (7.84) | 73.33 (7.53) | 0.284 |
| Sex – Male (n (%)) | 11 (68.8) | 352 (58.6) | 0.213 |
| Years Education (mean (SD) | 15.62 (2.58) | 16.15 (2.68) | 0.199 |
| APOE4 Count (n (%)) |  |  | 0.446 |
| 0 | 9 (56.2) | 334 (55.6) |  |
| 1 | 7 (43.8) | 215 (35.8) |  |
| 2 | 0 (0.0) | 52 (8.7) |  |
| Aβ (mean (SD) | 945.67 (350.97) | 1030.22 (445.41) | 0.211 |
| P-tau (mean (SD)) | 26.96 (14.41) | 26.92 (12.66) | 0.003 |
| Time in ADNI (mean (SD)) | 0.55 (1.44) | 0.58 (1.67) | 0.018 |

^a^ Standardized mean difference across groups

Table A3: Confounders stratified by presence of Asthma IV

|  | Asthma (n = 44) | No Asthma (n = 573) | SMD |
| --- | --- | --- | --- |
| Cardiovascular Issues – Yes (n (%) | 32 (72.7) | 406 (70.9) | 0.042 |
| Neurological Issues – Yes (n (%)) | 14 (31.8) | 200 (34.9) | 0.065 |
| Renal Issues - Yes (n (%)) | 17 (38.6) | 260 (45.4) | 0.137 |
| Age (mean (SD) | 70.21 (8.76) | 73.63 (7.39) | 0.422 |
| Sex – Male (n (%)) | 25 (56.8) | 338 (59.0) | 0.044 |
| Years Education (mean (SD) | 16.89 (2.22) | 16.08 (2.70) | 0.328 |
| APOE4 Count (n (%)) |  |  | 0.029 |
| 0 | 24 (54.5) | 319 (55.7) |  |
| 1 | 16 (36.4) | 206 (36.0) |  |
| 2 | 4 ( 9.1) | 48 ( 8.4) |  |
| Aβ (mean (SD) | 1096.83 (455.50) | 1022.74 (442.19) | 0.165 |
| P-tau (mean (SD)) | 26.10 (12.83) | 26.98 (12.69) | 0.069 |
| Time in ADNI (mean (SD)) | 0.48 (1.35) | 0.59 (1.68) | 0.073 |

Table A4: Confounders stratified by presence of GI disorder IV

|  | GI Disorder  (n = 149) | No GI Disorder (n = 400) | SMD |
| --- | --- | --- | --- |
| Cardiovascular Issues – Yes (n (%) | 130 (77.4) | 308 (68.6) | 0.199 |
| Neurological Issues – Yes (n (%)) | 71 (42.3) | 143 (31.8) | 0.217 |
| Renal Issues - Yes (n (%)) | 84 (50.0) | 193 (43.0) | 0.141 |
| Age (mean (SD) | 72.71 (7.83) | 73.64 (7.42) | 0.122 |
| Sex – Male (n (%)) | 95 (56.5) | 268 (59.7) | 0.064 |
| Years Education (mean (SD) | 16.04 (2.58) | 16.17 (2.71) | 0.048 |
| APOE4 Count (n (%)) |  |  | 0.197 |
| 0 | 103 (61.3) | 240 (53.5) |  |
| 1 | 56 (33.3) | 166 (37.0) |  |
| 2 | 9 (5.4) | 43 (9.6) |  |
| Aβ (mean (SD) | 1062.89 (430.84) | 1014.98 (447.49) | 0.109 |
| P-tau (mean (SD)) | 23.94 (10.50) | 28.03 (13.26) | 0.342 |
| Time in ADNI (mean (SD)) | 0.47 (1.38) | 0.62 (1.75) | 0.096 |

Figure A2 computes the bias ratio for the time since approval IV.^2^ A value greater than 1 implies that the 2SLS estimate of the treatment effect is more sensitive to an omitted confounder than the OLS estimate. Once again, if we assume that unobserved confounding is correlated with the observed confounder that we intentionally omit then we can make conclusions about each method's sensitivity to unobserved confounding. We can see that for many confounders, 2SLS with time since approval as an IV is more sensitive than OLS.

Figure A2: Plot of bias ratios for OLS vs. 2SLS sensitivity to omitted confounders


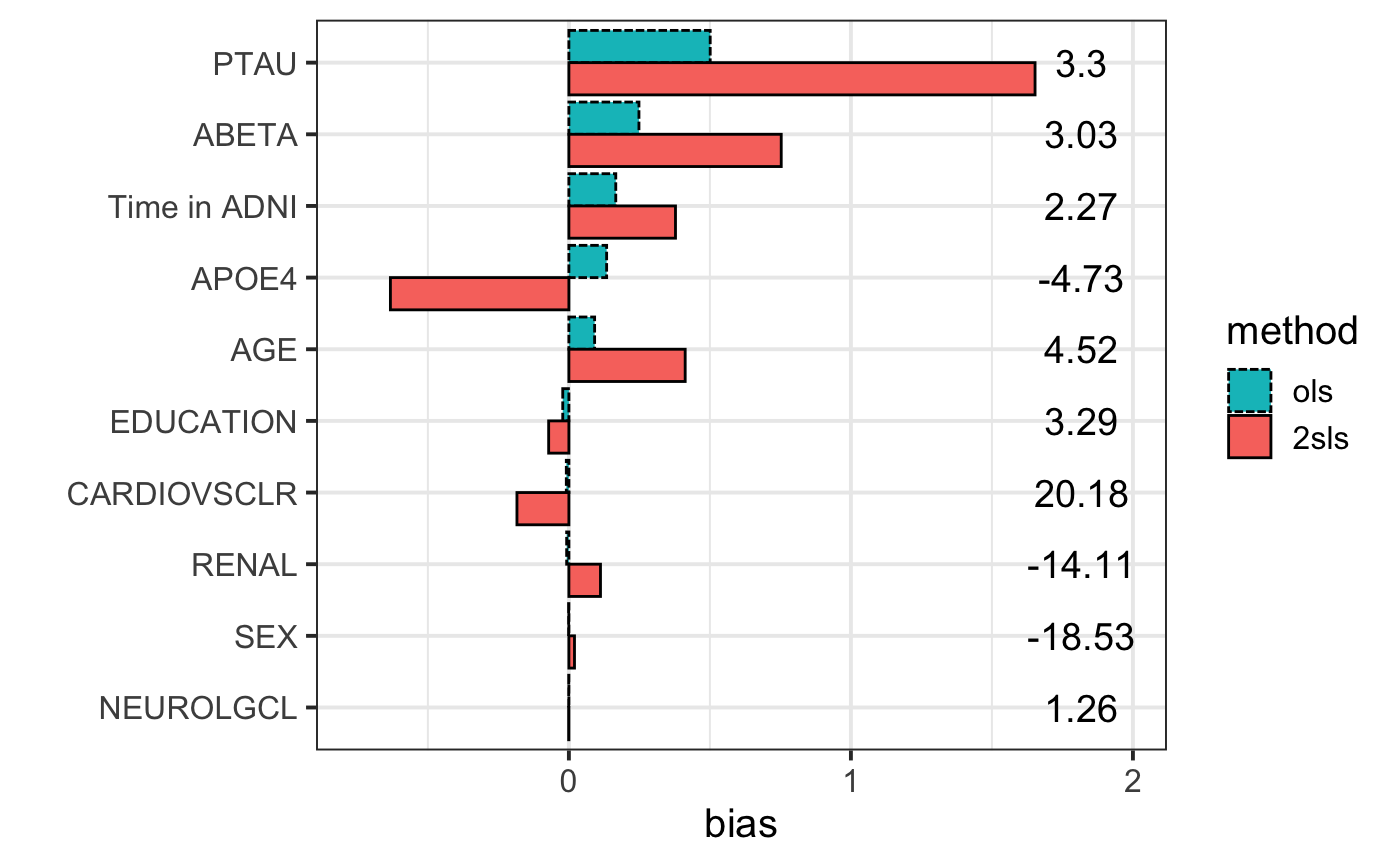


Section 2: Diagnostics related to IPTW

Table A5 presents post-matching balance and Figure A3 shows positivity. Note that these were computed on only one of the five imputed datasets. From the SMDs in Table A5, we see that propensity score weighting does well at balancing the confounders across the groups, especially the variables that were highly out of balance like time in ADNI and Aβ (see Table 3 in main paper).

Table A5: post matching balance of inverse probability weighted confounders

|  | Donepezil  (Effective Sample Size = 605.6) | Control  (Effective Sample Size = 616.6) | SMD |
| --- | --- | --- | --- |
| Cardiovascular Issues – Yes (n (%) | 418.4 (69.1) | 436.5 (70.8) | 0.037 |
| Neurological Issues – Yes (n (%)) | 201.2 (33.2) | 212.4 (34.5) | 0.026 |
| Renal Issues - Yes (n (%)) | 311.6 (51.5) | 281.1 (45.6) | 0.118 |
| Age (mean (SD) | 74.23 (7.25) | 73.44 (7.74) | 0.106 |
| Sex – Male (n (%)) | 329.8 (54.5) | 364.5 (59.1) | 0.094 |
| Years Education (mean (SD) | 15.98 (2.88) | 16.14 (2.73) | 0.057 |
| APOE4 Count (n (%)) |  |  | 0.124 |
| 0 | 370.3 (61.2) | 346.0 (56.1) |  |
| 1 | 179.0 (29.6) | 218.0 (35.4) |  |
| 2 | 56.2 (9.3) | 52.6 (8.5) |  |
| Aβ (mean (SD) | 1043.36 (456.52) | 1031.49 (441.21) | 0.026 |
| Time in ADNI (mean (SD)) | 0.76 (1.35) | 0.58 (1.79) | 0.113 |
| P-tau (mean (SD)) | 27.14 (12.32) | 26.87 (13.09) | 0.021 |

Figure A3 shows that overlap is adequate as, for the most part, each donepezil patient has a control patient with a similar propensity score except at the low and high propensity score ranges. There are many individuals in the control group with low propensity scores, which may give us problems related to near-positivity violations.

Figure A3: Overlap of predicted propensity scores by treatment group


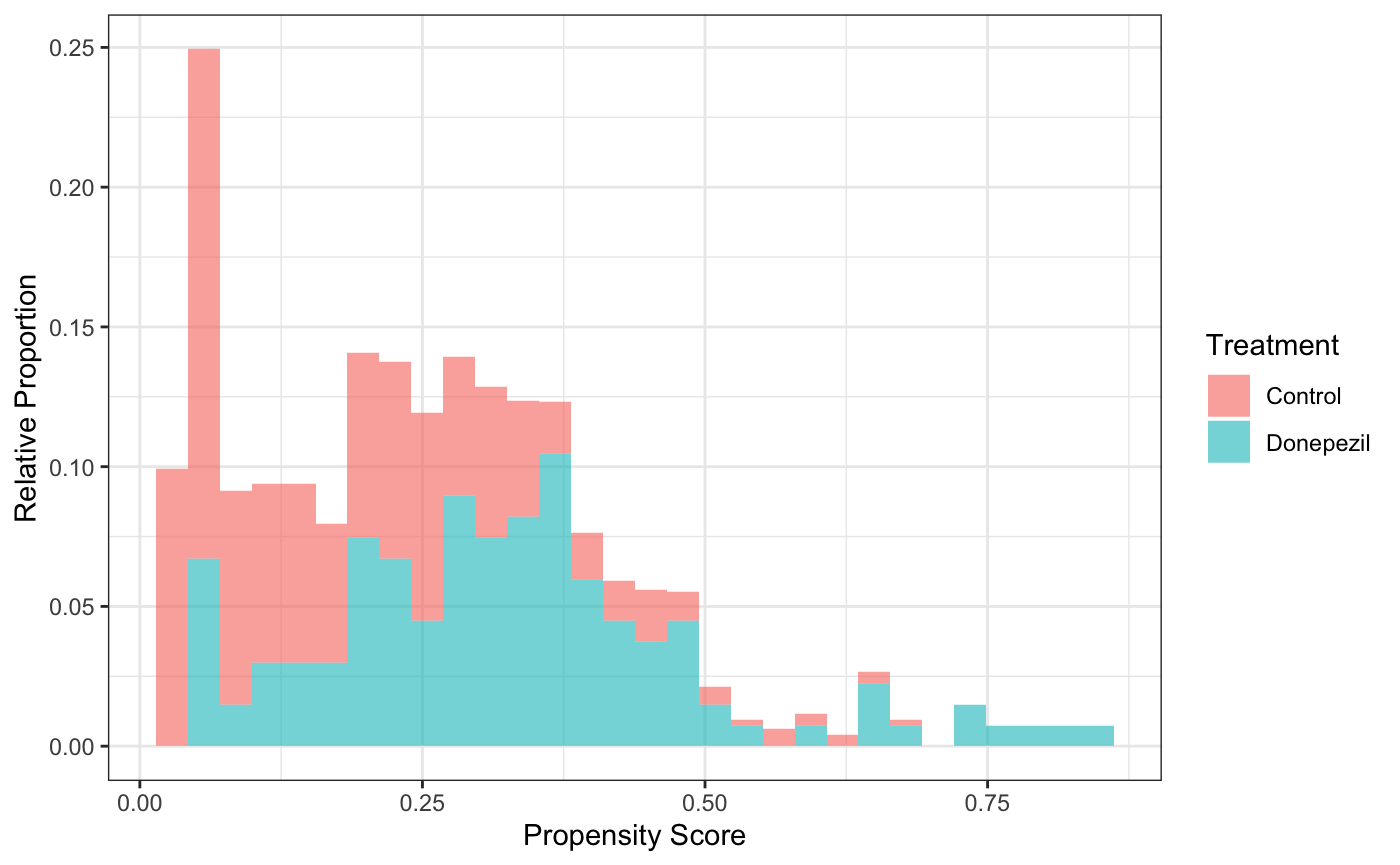


**References**

^1^Keele L, Small D. Instrumental variables: Don't throw the baby out with the bathwater. *Health Serv Res*. 2019 Jun;54(3):543-546.

^2^ Zhao Q, Small DS. Graphical diagnosis of confounding bias in instrumental variable analysis. *Epidemiology*. 2018 Jul 1;29(4):e29-31.
